# The Multi-faceted Ecto-enzyme CD38: Roles in Immunomodulation, Cancer, Aging, and Metabolic Diseases

**DOI:** 10.3389/fimmu.2019.01187

**Published:** 2019-05-31

**Authors:** Kelly A. Hogan, Claudia C. S. Chini, Eduardo N. Chini

**Affiliations:** Signal Transduction and Molecular Nutrition Laboratory, Kogod Center on Aging, Department of Anesthesiology and Perioperative Medicine, Mayo Clinic College of Medicine, Rochester, MN, United States

**Keywords:** CD38, NADase, NAD+, aging, cancer, metabolism, senescence, macrophages

## Abstract

CD38 (Cluster of Differentiation 38) is a multifunctional ecto-enzyme that metabolizes NAD+ and mediates nicotinamide dinucleotide (NAD+) and extracellular nucleotide homeostasis as well as intracellular calcium. CD38 is also an emerging therapeutic target under conditions in which metabolism is altered including infection, aging, and tumorigenesis. We describe multiple enzymatic activities of CD38, which may explain the breadth of biological roles observed for this enzyme. Of greatest significance is the role of CD38 as an ecto-enzyme capable of modulating extracellular NAD+ precursor availability: 1 to bacteria unable to perform de novo synthesis of NAD+; and 2 in aged parenchyma impacted by the accumulation of immune cells during the process of ‘inflammaging’. We also discuss the paradoxical role of CD38 as a modulator of intracellular NAD+, particularly in tumor immunity. Finally, we provide a summary of therapeutic approaches to CD38 inhibition and ‘NAD+ boosting’ for treatment of metabolic dysfunction observed during aging and in tumor immunity. The present review summarizes the role of CD38 in nicotinamide nucleotide homeostasis with special emphasis on the role of CD38 as an immunomodulator and druggable target.

## Introduction

Historically, CD38 was considered a surface marker used to identify T cells (1). Our discovery that CD38 is the main nicotinamide dinucleotide (NAD^+^) catabolic enzyme has shed light on the relevance of this enzyme in organismal NAD^+^ metabolism and has implications for several pathophysiological conditions including infection, aging, tumorigenesis (2–6). Furthermore, a more sophisticated understanding of the structure and function of CD38 has led to identification of therapeutic targets and potential treatments for conditions associated with metabolic dysfunction in infection, aging, and cancer (7–10). The role of CD38 in other organ systems, such as the central nervous system, has also been described. These studies focus on oxytocin signaling and social behavior in mice (11–14) and have been reviewed previously (15). Thus, the present review will provide an overview of CD38 enzymology; focus on recent advances in CD38-mediated age-related metabolic dysfunction and tumor immunometabolism; and summarize pharmacological approaches to CD38 inhibition.

### The Different Enzymatic Activities of CD38 Provide Diversity in Its Biological Roles

The surface marker and multifunctional enzyme CD38 appears to provide a link between inflammation and age- and disease-related decline in tissue homeostasis and, therefore, represents a critical target for therapeutic intervention. CD38 is expressed predominately on immune cells in response to stimulation by cytokines, endotoxins, and interferon (16–18). Expression of the enzyme is regulated by a promoter region containing binding sites for NF-kB, RXR, LXR, and STAT suggesting that it plays a key role in the inflammatory response (18). CD38 expression causes a substantial decline in cellular NAD^+^ levels, thus altering the availability of substrates for enzymes regulating cellular homeostasis. Thus, infiltration of CD38-expressing immune cells during infection, aging, or tumorigenesis has the potential to: alter NAD^+^ homeostasis in parenchymal tissues or the tumor microenvironment; disrupt normal metabolic processes; and undermine tissue integrity.

**Figure 1:**
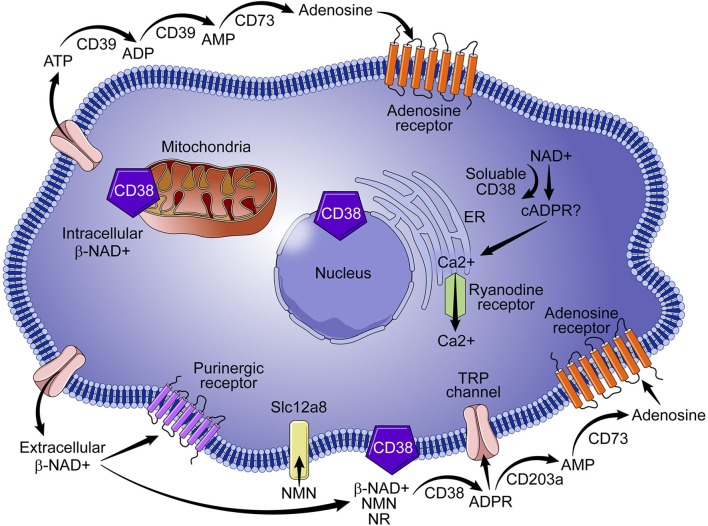
Role of CD38 in NAD^+^ metabolism. CD38 is predominantly expressed on immune cells and metabolizes nicotinamide nucleotides (NAD^+^ and NMN) to ADPR and cADPR, which results in the mobilization of calcium. Although intracellular CD38 is present in the cytoplasm and in the membranes of organelles, a vast majority of CD38 activity is extracellular, which results in degradation of NAD^+^ precursors (e. g., NMN) necessary for NAD^+^ synthesis. Extracellular activity of CD38 has wide ranging implications for NAD^+^ homeostasis in the context of infection, metabolic dysfunction, aging, and tumor biology.

The identification of CD38 as a key modulator of NAD^+^ metabolism in the context of cell signaling, aging, and tumor biology suggests that the enzyme is a target of promising therapeutic potential. CD38 is paradoxical in its mode of action, diverse in its locale, and functionally pleotropic, and thus presents numerous drug design challenges and opportunities. CD38 is positioned in the cellular membrane with its catalytic site facing toward the extracellular environment in a type II orientation (Figure 1) (19–21). Functionally, over 90% of CD38 acts as an ecto-NADase that catabolizes β-NAD^+^ (20). Given the abundance of intracellular NAD^+^, the presence of an extracellular catalytic site presents a topological paradox (19, 21). Our laboratory and others demonstrate that, in addition to NAD^+^, CD38 metabolizes extracellular NAD^+^ precursors (NMN and NR) prior to their transport into the cell for NAD^+^ biosynthesis (5). We have shown that the ecto-NMNase activity of CD38 plays a critical role in the regulation of nicotinamide nucleotides during the aging process (5). These findings suggest that the role of type II membrane-bound CD38 is to maintain NAD^+^ homeostasis by regulating precursors of NAD^+^ synthesis in the extracellular environment, thus calling into question the veracity of a topological paradox. Another outstanding question in the field is the role of intracellular forms of CD38. As discussed above, a small percent of CD38 can have its catalytic site facing the intracellular environment. Examples include the transmembrane Type III CD38 that has its c-terminal facing intracellularly; CD38 present in the nucleus and mitochondrial membrane; and a likely soluble form of CD38 present in the cytoplasm (Figure 1). In these configurations CD38 would have access to the intracellular pools of NAD^+^ and, without significant regulation, could lead to a severe decline in intracellular NAD^+^ and result in metabolic collapse (7, 19, 21, 22).

**Figure 2:**
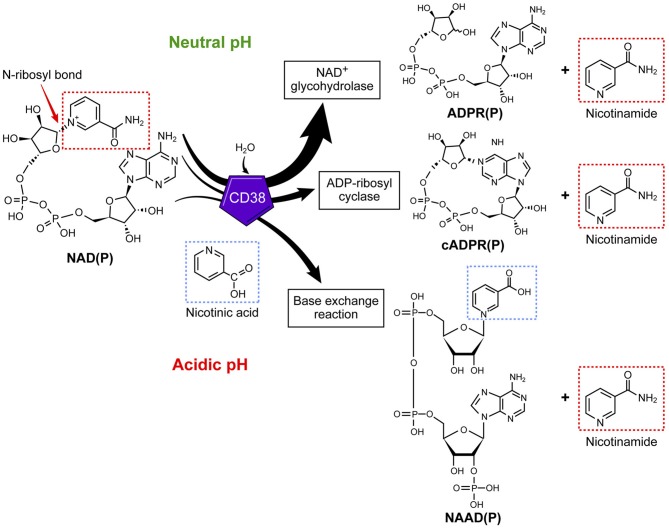
CD38 is primarily an NAD^+^ glycohydrolase, which degrades nicotinamide nucleotides. In addition to the NAD^+^ glycohydrolase activity, CD38 exhibits inefficient ADP-ribosyl cyclase activity and mediates a base-exchange of vitamin B3 bases in the presence of excess nicotinamide analogs.

The role of CD38 as a second messenger enzyme for the synthesis of cADPR is also proposed (Figure 1) (21–24). Interestingly, CD38 is a very inefficient cyclase and must degrade nearly 100 molecules of NAD^+^ to generate one molecule of cADPR (Figure 2) (7). It is possible that the generation of cADPR could be part of a byproduct of the NAD^+^ glycohydrolase activity of CD38. Furthermore, the structurally unrelated enzyme SARM1 has NAD^+^ glycohydrolase activity and generates a very small fraction of cADPR during its catalysis (25). This may indicate that the NADase activity of SARM also generates cADPR as a byproduct. Thus, the widely accepted notion that CD38 is mainly an ADP-ribosyl cyclase is misleading based on the fact that its primary role is to degrade nicotinamide nucleotides such as NAD^+^ and NMN. Therefore, we propose that the main enzymatic activity of CD38 is not an ADP-ribosyl cyclase, but rather an NAD^+^ glycohydrolase (Figure 2) and that both CD38 and SARM1 should be characterize based on their main enzymatic activity, namely the NAD/NMNase. In spite of these enzyme classifications, it is clear that cADPR is a second messenger that regulates intracellular calcium homeostasis in several cells (Figure 1) (24, 26, 27).

Another role for CD38 is the regulation of extracellular adenosine, which requires consumption of NAD^+^. The synthesis of adenosine from NAD^+^ is a complementary mechanism similar to the CD39/CD73-mediated catabolism of ATP to adenosine (Figure 1) (28, 29). Adenosine is important in immune modulation because it has been implicated in immune suppression through purinergic receptor binding (Figure 1) and in the immunomodulation of multiple myeloma (28, 29) and lung cancer (30). Thus, involvement of CD38 in the regulation of NAD^+^ and adenosine homeostasis has led to some speculation that CD38 may function as an immune check point molecule (31, 32).

In addition to the NAD^+^ glycohydrolase and ecto-NMNase activity of CD38, the enzyme catalyzes the base-exchange reaction that leads to an exchange of vitamin B3 bases on a molecule (Figure 2). This reaction is optimal in the presence of excess nicotinamide analogs and at low pH; however, this reaction can also be catalyzed at physiological pH (33–35). One of the potential molecules that can be generated by this reaction is the nicotinic acid derivative of NADP (NAADP) (Figure 2) (36). Whether this reaction occurs in cells naturally expressing CD38 or *in vivo* is unknown; however, the synthesis of NAADP by CD38 by a base-exchange reaction in lysosomes would have implications for intracellular calcium homeostasis. Interestingly, NAADP levels in tissues and cells appear to be independent of the CD38 expression (33–36), indicating that CD38 independent NAADP synthesis exists in mammalian tissues. One of the potential candidates for the synthesis of NAADP is the newly discovered NADase SARM1.

In any case, it appears that the base-exchange reaction operates *in vivo* when excess nicotinamide analogs are available to tissues (Figure 2) (37). For example, we have demonstrated that CD38 is responsible for the synthesis of isoniazid derivatives of NAD^+^ and NADP^+^ in animals given toxic doses of this anti-tuberculosis medication (37). Isoniazid is a nicotinamide derivative that can serve as a substrate for the CD38-mediated base-exchange reaction (37). Thus, it is possible that the CD38 base-exchange reaction is partially responsible for the *in vivo* toxicity of isoniazid through the formation of toxic NAD^+^ intermediates during drug metabolism. It is important to highlight that, in addition to its enzymatic activity, CD38 could have enzymatic-independent roles in cell migration and homing through interaction with adhesion molecules such as CD31 (38).

### CD38 Has a Role in the Immune Response to Microbes

**Figure 3:**
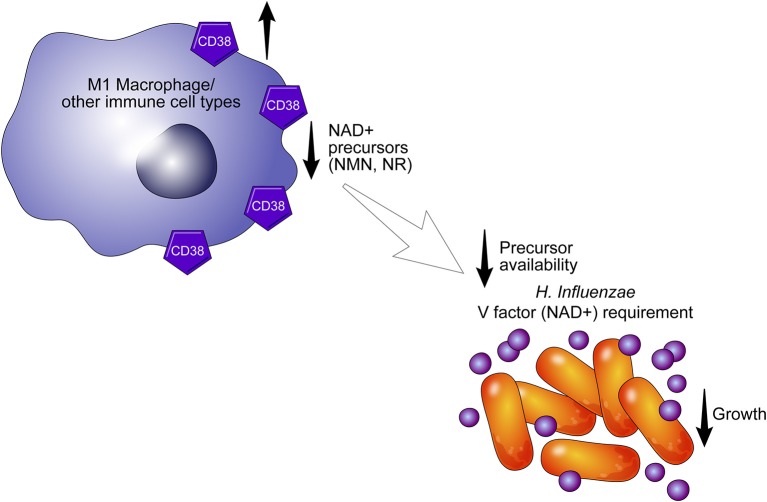
CD38 may alter the availability of NAD^+^ precursors. CD38+ cells may limit the growth of pathogens such as *H. aegyptius, H. influenzae, H. haemolyticus, H. parainfluenzae*, and *H. parahaemolyticus* by degrading extracellular NAD^+^ precursors required for NAD^+^ synthesis in bacteria. In the absence of NAD and its precursors (V factor), pathogens undergo metabolic collapse.

An important and still outstanding question is why inflammatory cells express CD38. The inability of bacteria such as *H. influenza* to recycle or perform *de novo* synthesis of NAD^+^ may provide insight into the role of CD38 in response to infection (39, 40). These pathogens, including *H. aegyptius, H. influenzae, H. haemolyticus, H. parainfluenzae, and H. parahaemolyticus*, are responsible for a spectrum of infections, and lack the ability to synthesize NAD^+^ and rely instead on uptake of NAD^+^ and NAD^+^ precursor molecules (e. g., NMN, NR) to support metabolism and growth (40). In fact, NAD^+^ and its precursors are necessary for the growth of bacteria and have to be included in culture media as the V-factor (Figure 3) (39). Interestingly, these bacteria express nucleotide transporters to facilitate intracellular incorporation (40). The presence of CD38 ecto-nicotinamide nucleotidase in activated immune cells may reduce the availability of NAD^+^ to bacteria and other pathogens and could limit the development or progression of infections. This process may occur not only extracellularly but also in the intracellular space. Indeed, we have demonstrated previously that CD38^+^ macrophages limit the growth of some obligatory intracellular bacteria (18). We propose that CD38, as either an ecto-enzyme or as an intracellular enzyme, promotes metabolic collapse in pathogens by degrading NAD^+^ and its precursors.

In addition to macrophages, which express CD38 in M1 polarization (18, 41, 42), others have reported a role for CD38 in neutrophil- and T cell-mediated immune response (43, 44). Neutrophils lacking CD38 demonstrate altered mobilization from the bone marrow and migration to sites of infection (43, 45, 46). CD38^+^ T cells play a myriad of roles in acute and chronic infections including the ability to be cytotoxic (47) as well as inhibit an immune response (48). What remains unknown is whether some effects are mediated by the enzymatic or non-enzymatic roles of CD38. Taken together, expression of CD38 on immune cells appears to play a role in the immune system, particularly in the context of infection.

### CD38 in Aging and Age-Related Metabolic Dysfunctions

**Figure 4:**
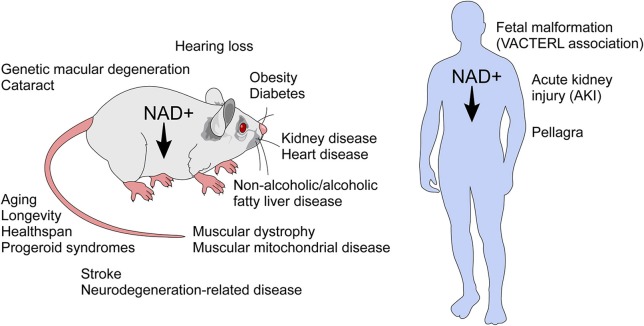
NAD^+^ decline has been implicated in several diseases and age-related conditions. NAD^+^ decline has been implicated in the biology of aging and in several conditions in rodents. In humans, NAD decline has been implicated in pellagra, acute kidney injury, and the fetal malformation syndrome (VACTERL association), which affects many organ systems during development.

Unlike an immune response to infection, inflammaging is a “sterile” inflammatory response which is cytokine-mediated and prompted by damage to DNA and proteins as well as a diminished capacity for cell repair in the aging organism (49, 50). In age-related decline, there is a reduction of NAD^+^, a master regulator of metabolism, which when reduced is a cofactor in electron transport during oxidation-reduction reactions. Furthermore, NAD^+^ is a critical molecule in cell signaling, intracellular calcium regulation, and chromatin remodeling (24, 26, 27, 36, 43, 51–53). NAD^+^ has emerged as a molecule that provides a link between signaling and metabolism. Decline of NAD^+^ levels is very likely a key player in the pathogenesis of several diseases including age-related conditions (Figure 4) (4–6, 9, 52, 54–61).

Until recently, age-related NAD^+^ decline was thought to be a consequence of activation of NAD^+^-consuming DNA-repair enzymes such as Poly-ADP-ribosylating enzymes (PARPs) or decrease in NAD^+^ synthesis (9, 62). Unlike CD38, PARPs are found exclusively in the intracellular compartment, more specifically in the nuclei (9). PARPs are activated by DNA damage and catalyze the NAD-dependent polymerization of a chain of polymeric adenosine diphosphate ribose (poly (ADP-ribose) or PAR, which signals repair of DNA. PARPs lead to intranuclear and intracellular NAD^+^ consumption and NAD^+^ collapse (9). Additionally, levels of the rate-limiting NAD^+^ synthesis enzyme NAMPT decrease in some aging tissues (9, 63, 64) indicating a diminished capacity to generate intracellular NAD^+^ by salvage pathway synthesis and reliance on import of extracellular NAD^+^ precursors NMN and NR.

**Figure 5:**
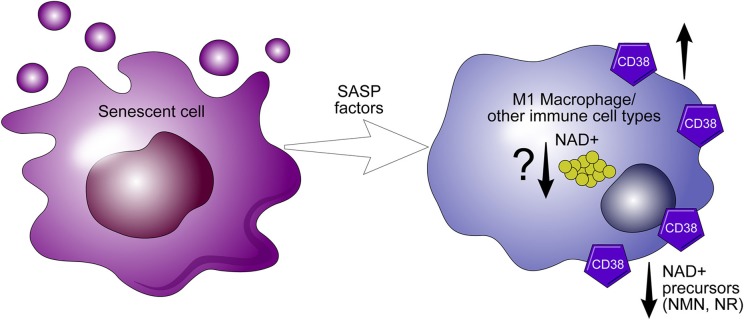
CD38 expression in macrophages mediate “inflammaging.” The senescence associated senescent phenotype (SASP) appears to drive the expression of CD38 in macrophages. During inflammaging, CD38+ macrophages accumulate in aging tissues and potentiate the decline of precursors necessary for NAD^+^ synthesis.

We have shown that genetic ablation of CD38 protects against age-related NAD^+^ decline and mitochondrial dysfunction. Furthermore, levels and activity of CD38 increase during aging (5). Recently, we proposed that NAD^+^ decline during aging in cells/tissues is related to exposure to factors related to the senescence associated secretory phenotype (SASP) which may increase CD38 expression in these cells/tissues. In fact, we demonstrated that SASP conditioned media from senescence cells can induce CD38 expression in both macrophages and endothelial cells (65). Thus, it is possible that the senescent phenotype drives the accumulation of CD38^+^ inflammatory cells, which modulate the availability of NAD^+^ precursors, and plays a crucial role in the metabolism of nicotinamide nucleotide (Figure 5) (65).

### CD38 in Immune Metabolism and Tumor Biology

Age-related diseases such as cancer provide an interesting context for considering ways in which immune cells are modulated by the tumor microenvironment as well as targeted by immune-based therapies. Emerging data demonstrate that CD38 knockout mice under high metabolic pressure, such as high fat diets, are protected against the development of cancers and have increased longevity (62). Interestingly, the role of CD38 in the tumor cell provides somewhat conflicting data. For example, pancreatic and prostate cancer, which exhibit low CD38 expression and increased cellular NAD^+^ levels, exhibit increased tumor cell survival (66, 67). Increased CD38 activity in both adenocarcinomas, results in decreased intracellular NAD^+^, diminished cell growth, and increased apoptosis and cellular senescence (66–68).

On the other hand, metabolic reprogramming of NAD^+^ regulation via inhibition of CD38 has been proposed as a strategy for improving efficacy of immune-based therapies. CD38, in particular, appears to play a significant role in the regulation of metabolism and immunomodulation of the tumor microenvironment (1, 31, 38, 69–76).

CD38 has emerged recently as a component of mitochondrial transfer/trafficking between cells (77, 78). For many years, it has been demonstrated that cells can transfer their mitochondria to other cells, particularly cancer cells (79–84). However, components of this intriguing biological process have not been identified. In particular, it has been shown that multiple myeloma cells may take or receive mitochondria from non-malignant bone marrow stromal cells. This transfer occurs via a CD38-dependnet tumor-derived tunneling nanotubes (78). The precise enzymatic, structural, and signaling roles for CD38 in this process have not been described.

### CD38 Regulates Anti-Tumor T Cell Exhaustion

**Figure 6:**
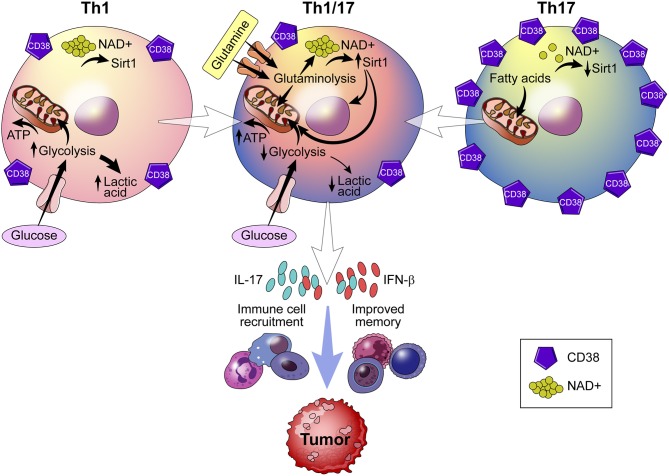
Role of CD38 in immunometabolism and tumor biology. Using a strategy that merges Th1 and Th17 cells into a hybrid Th1/Th17 cell, the CD38-NAD^+^ axis undergoes metabolic reprogramming. Reprogramming results in a shift toward glutamine-driven oxidative phosphorylation, which improves T cell survival, immune cell recruitment to the tumor, and T cell memory. Th1/Th17 hybrid cells also demonstrate reduced expression of CD38 and higher levels of NAD^+^, which serves as a substrate for NAD^+^ -dependent Sirt1 deacetylase activity that is essential to the T cell response.

Recently, metabolic reprogramming of the CD38-NAD^+^ axis in CD4^+^ T cells has shown to be a promising approach to adoptive T cell (ACT) therapy (75). ACT therapy involves the *ex vivo* alteration, expansion, and transfer of tumor epitope reactive autologous T cells to a tumor bearing host. What results is a population of T cells with enhanced anti-tumor potential. The dependence of CD4^+^ T cells on an array of metabolites deems this population a particularly robust target for metabolic reprogramming (85). Chatterjee et al. report a strategy in which Th1 and Th17 cells are merged into hybrid Th1/17 cells, which rely on glutamine-driven oxidative phosphorylation (glutaminolysis) (75). This shift toward glutaminolysis confers greater survival of Th1/17 cells, elevated secretion of IFN-γ and IL-17 production, and enhanced anti-tumor activity (Figure 6). Curiously, Th1/17 cells demonstrate robust activity of the transcription factor Foxo1, which controls T cell memory and is responsible for the anti-tumor phenotype of the Th1/17 hybrid cell type. Induction of Foxo1 occurs by deacetylation by NAD^+^-dependent Sirt1 and is essential to the immune response of T cells. Chatterjee et al. demonstrate that, in addition to increased Foxo1 activity, Th1/17 exhibit higher levels of NAD^+^ and reduced CD38 expression (75). The significance of this finding is that increased NAD^+^ levels improve the anti-tumor response of Th1/17 by providing a substrate for Sirt1. Chatterjee observed a similar phenotype in CD38-deficient natural killer (NK) cells, regulatory T cells (Tregs), and myeloid-derived suppressor cells (MDSCs) (75). What remains unknown, however, is how glutaminolysis controls production of NAD^+^ and whether CD38 inhibition is a function of glutamine catabolism.

### CD38: A New Immune Check Point?

Programmed death-ligand 1 (PD-L1, B7-H1, or CD274) is present on the surface of dendritic cells or macrophages, but is also frequently over-expressed in tumor cells. Tumor cells co-opt the PD-L1 in order to evade T cell surveillance (86). CD8^+^ cytotoxic T cells treated with checkpoint inhibitors such as PD-1/PD-L1 blocking antibodies demonstrate temporal upregulation of CD38 (87). Increased CD38 activity, in turn, results in CD8^+^ T cell suppression (73). This phenomenon may in part explain the high drug resistance rates observed in patients treated with PD-1/PD-L1 blockers (88). The mechanism by which CD38 mediated PD-1/PD-L1 blockade resistance likely includes perturbation of adenosine receptor signaling in tumor microenvironment leading to immune modulation (87). CD38 expression in tumors is a biomarker of poor response to checkpoint inhibitor therapy (88). One possible approach to ameliorate resistance to PD-1/PD-L1blockade is co-inhibition of CD38, which may re-establish the immune response of T cells to the tumor. Taken together, CD38 inhibition is emerging as a potential therapeutic strategy for shaping the immunometabolome of host T cells in order to enhance immune response to tumor cells.

### CD38 in Fetomaternal Tolerance

Successful pregnancy requires the establishment of maternal immune tolerance to the fetus (89). For the uterine environment to be receptive to pregnancy, there must be activation, differentiation, and expansion of toleragenic dendritic cells (tDCs) and CD4^+^CD25^+^ Treg cells (90, 91) Evidence from a CBAxDBA/2 mouse model of pregnancy failure suggests that components of seminal fluid prime the immunological landscape of the uterine environment for implantation (92). Several years ago our group demonstrated that human seminal fluid contains high amounts of a soluble 49 kDa CD38 enzyme (93). Interestingly, the glycohydrolase activity of CD38 in the seminal fluid is significantly inhibited by the high zinc content of the seminal fluid (93). These data raise the possibility that seminal fluid CD38 could control the metabolic fate of NAD^+^ and adenosine and regulate sperm and fetomaternal tolerance. A study by Kim and colleagues demonstrated that in matings between CD38^−/−^ male mice and wild type females, embryo reabsorption rates are higher, and fewer total and viable implantations sites are observed suggesting a role for CD38 in establishing pregnancy (94). Indeed, tDCs and Foxp3^+^ Tregs were induced by soluble seminal CD38 through a CD31- independent pathway (94). These data indicate that CD38 may play a similar role in fetomaternal tolerance in humans and further highlight the physiological role of CD38 in immunomodulation. What remains poorly understood, however, is the specific mechanism of CD38 enzymatic activity at the fetomaternal tolerance. For example, it is not known if CD38-mediated regulation of NAD^+^ metabolism or the accumulation of adenosine is involved in establishing tolerance between mother and fetus.

## Pharmacological Approaches to Targeting/Modulating CD38 Ecto- and Endo Enzymatic Activity

Based on the multi-faceted roles of CD38, there are extensive opportunities for design of molecules capable of inhibiting this enzyme. Below we summarize those molecules and discuss their potential as immunomodulatory therapeutics.

### Anti-CD38 Antibodies

Anti-CD38 monoclonal antibodies are shown to be highly efficacious in the treatment of multiple myeloma (MM) and pre-clinical studies highlight the potential use in other tumors such as CLL, lung cancer, prostate cancer and melanoma (29, 70, 72–74, 76, 95–99) and in a preclinical model of melanoma (87). Currently, there are four monoclonal antibodies in clinical trials for the treatment of CD38^+^ malignancies (76, 95, 96). These include Daratumumab (Janssen Biotech) (31, 32, 66, 72, 74, 76, 95–97), Isatuximab (Sanofi-Aventis) (73, 76, 98), MOR202 (Morphosys) (76), and TAK079 (Takeda) (100) (Table 1), which produce antibody-dependent cell-mediated toxicity (ADCC) and have comparable binding affinities and similar safety profiles. These antibody-based therapies, however, differ in their ability to induce direct apoptosis, mount complement-mediated cytotoxicity (CDC), inhibit CD38 directly, and induce antibody-dependent cell-mediated phagocytosis (ADCP) (Table 1) (76, 96). The prevailing mechanism of action of anti-CD38 antibodies currently under investigation is hypothesized to be ADCC; however the therapeutic benefit may also involve inhibition of CD38 NADase activity and subsequent NAD^+^ boosting. Recently, Chatterjee et al. raised the possibility that T cell function in the tumor microenvironment can be enhanced by inhibition of CD38 and increased availability of NAD^+^ (75). This finding supports the tandem use of anti-CD38 antibodies and PD-1/PD-L1 checkpoint blockers to enhance the immune response to cancer, thereby providing a two-pronged approach to immunomodulation of the tumor microenvironment.

**Table 1:** Therapeutic approaches to CD38 inhibition and immunomodulation.

		**Compound name**	**Mechanism of action**
NAD analogs	Covalent inhibitors	Ara-F-NAD	Competitive inhibition of NADase activity
		Ara-F-NFM	
		Ara-F-NMN phosphoester/C48	
	Non-covalent inhibitors	Carba-NAD	Competitive inhibition of NADase activity
		Pseudo-Carba-NAD	
		Apigenin	
Flavonoids		Luteolinidin	
		Kuromanin	
		Rhein/K-Rhein	Uncompetitive inhibition of NADase activity
4-amino- quinolines		78c	Uncompetitive inhibition of NADase activity
		1ah	Competitive inhibition of NADase activity
		1ai	
Antibodies (IgG mAB)		Isatuximab	Allosteric inhibition of NADase activity/Cytotoxic effect/clearance of CD38+ cells
		Daratumumab	Cytotoxic effect/clearance of CD38+ cells
		TAK-079	
		MOR-202	

*NAD^+^ analogs, flavonoids, small molecule inhibitors (4-amino-quinolines), and antibodies have been shown to have therapeutic potential as immunomodulators in conditions of aging and tumor immunity*.

### Small Molecule CD38 Inhibitors and Derivatives of 4-Amino-Quinoline

There are over 200 compounds capable of inhibiting CD38 (Table 1). These compounds are classified as NAD analogs (101), flavonoids (102), and heterocyclic compounds (103, 104) and can form either covalent or non-covalent bonds to amino acids located in the active site of CD38 (105). Elucidation of CD38 catalysis and active site crystallography has resulted in design of high affinity CD38 small molecule inhibitors (26, 103, 106). Small molecule derivatives of 4-amino-quinoline, in particular, are non-covalent and demonstrate an IC50 values in the nanomolar range (103, 104) and include three lead compounds: 78c (IC50 7.3 nM), 1ai (IC50 46 nM), and Iah (IC50 115 nM). Mutagenesis studies implicate binding site residues that are important for NADase activity (105). Changes to these residues result in loss of glycohydrolase activity and increased cyclase activity. Small molecule blockade of CD38, therefore, compromises interactions between endogenous substrates (NAD^+^, NMN) and the active site. CD38 small molecule inhibitors show promise as NAD^+^ boosting drugs in pre-clinical studies (103, 104, 106) and are likely to be important tools for understanding disease-specific roles for the multi-functional enzyme.

### Flavonoids

Several naturally occurring compounds are reported to inhibit the catalytic activity of CD38 including flavonoid compounds apigenin, quercetin, and leteolinidin (8, 102, 107) (Table 1). Most flavonoids, with the exception of 4,5-dihydroxyanthrquinone-2-carboxylic acid (RHein), are competitive antagonists of CD38, which likely lead to an increase in cellular NAD^+^ levels and activation of NAD-dependent enzymes such as SIRTUINs (8). Flavonoid CD38 inhibitors demonstrate a lack of toxicity in humans (8, 102) and beneficial effects in animal models of obesity, heart ischemia, kidney injury, viral infection, and cancer (8, 42, 71, 102, 107, 108). However, these compounds lack specificity and demonstrate off-target effects and a poor oral pharmacokinetic profile (8, 102).

### NAD^+^ Analog Inhibitors

Based on the mechanisms of CD38 catalysis and crystal structure, NAD^+^ analog inhibitors were developed by modifying nicotinamide ribose group within NAD^+^ and NMN (101, 109–111). These inhibitors include the low affinity, non-covalent inhibitor carba-NAD and ara-NAD (Table 1). Although these drugs may have potential as immunomodulators capable of increasing tumor suppressive effects (75), their utility may be limited by their inhibitory effects on NAD^+^ consuming enzymes.

## Concluding Remarks

CD38 has emerged as a major NAD^+^ consuming enzyme and as a regulator of NAD^+^ homeostasis in several conditions. The primary role of CD38 in immunity has not been completely understood. However, it is possible that CD38 may have a key role in the immune response to bacterial infections. It has also been described that the CD38-NAD^+^ axis controls metabolic reprogramming of T cells in the tumor microenvironment and contributes to drug resistance plaguing PD-1/PD-L1checkpoint inhibitors. As a central regulator of metabolism, maintenance of NAD^+^ homeostasis is taking center stage as a modifier of health and disease. These findings highlight the potential drugability of CD38 as a target in metabolic dysfunction typically observed in the contexts of aging and tumor immunity. In addition, CD38 cytotoxic antibodies has been used to treat CD38 positive tumors such as multiple myeloma and potentially CLL. Thus, CD38 has both physiological and pathological roles as well as great potential as a therapeutic target in various human diseases.

## Author Contributions

All authors listed have made a substantial, direct and intellectual contribution to the work, and approved it for publication.

## Conflict of Interest Statement

EC has a patent on CD38 inhibitors and is a consultant for Teneobio. The remaining authors declare that the research was conducted in the absence of any commercial or financial relationships that could be construed as a potential conflict of interest.

## Abbreviations

NAD^+^: Nicotinamide adenine dinucleotide
NMN: Nicotinamide mononucleotide
NR: Nicotinamide riboside
NAADP: Nicotinic acid adenine dinucleotide phosphate
ADP: Adenosine diphosphate
ADPR: ADP-ribose
cADPR: cyclic ADP-ribose
NADP^+^: Nicotinamide adenine dinucleotide phosphate
SARM1: Sterile alpha and TIR Motif Containing 1
VACTERL, Vertebral defects, trachea-esophageal fistula, renal anomalies: and limb abnormalities
RXR: Retinoid X receptors
LXR: Liver X receptor
STAT: Signal transducer and activator of transcription
PARP: Poly ADP ribose polymerase
PD-1: Programmed cell death protein 1
PD-L1: Programmed death ligand 1.
